# Co-infection with SARS-CoV-2 and Influenza A Virus in Patient with Pneumonia, China

**DOI:** 10.3201/eid2606.200299

**Published:** 2020-06

**Authors:** Xiaojing Wu, Ying Cai, Xu Huang, Xin Yu, Li Zhao, Fan Wang, Quanguo Li, Sichao Gu, Teng Xu, Yongjun Li, Binghuai Lu, Qingyuan Zhan

**Affiliations:** China-Japan Friendship Hospital, Beijing, China (X. Wu, Y. Cai, X. Huang, X. Yu, L. Zhao, S. Gu, B. Lu, Q. Zhan);; The Sixth Medical Center of PLA General Hospital, Beijing (F. Wang);; Weifang No. 2 People’s Hospital, Weifang, China (Q. Li);; Vision Medicals Co., Ltd., Guangzhou, China (T. Zu, Y. Li)

**Keywords:** 2019 novel coronavirus disease, SARS-CoV-2, COVID-19, severe acute respiratory syndrome coronavirus 2, influenza A, co-detection, viruses, China, influenza, zoonoses, co-infection, pneumonia

## Abstract

We report co-infection with severe acute respiratory syndrome coronavirus 2 (SARS-CoV-2) and influenza A virus in a patient with pneumonia in China. The case highlights possible co-detection of known respiratory viruses. We noted low sensitivity of upper respiratory specimens for SARS-CoV-2, which could further complicate recognition of the full extent of disease.

In December 2019, a series of cases of pneumonia of unknown cause was reported in Wuhan, Hubei Province, China. On January 7, 2020, the causative pathogen was identified as a virus subsequently named severe acute respiratory syndrome coronavirus 2 (SARS-CoV-2) (*1*–*3*). We report a case of co-infection with SARS-CoV-2 and influenza A virus in China.

A 69-year-old man was seen in the clinic of China-Japan Friendship Hospital on January 23, 2020, for fever and dry cough. The patient visited Wuhan from December 18, 2019–January 22, 2020, and began having symptoms January 23. He reported no underlying medical conditions. Routine blood tests revealed a leukocyte count of 5.70 × 10^9^ cells/L (reference range 3.5–9.5 × 10^9^ cells/L) and lymphocyte count of 2.18 × 10^9^ cells/L (reference range 1.1–3.2 × 10^9^ cells/L). Chest computed tomography revealed a mass, ground-glass consolidation in the right inferior lobe of the lungs (Figure, panel A). Because of the patient’s travel history, he was isolated for suspected coronavirus disease (COVID-19). 

**Figure:**
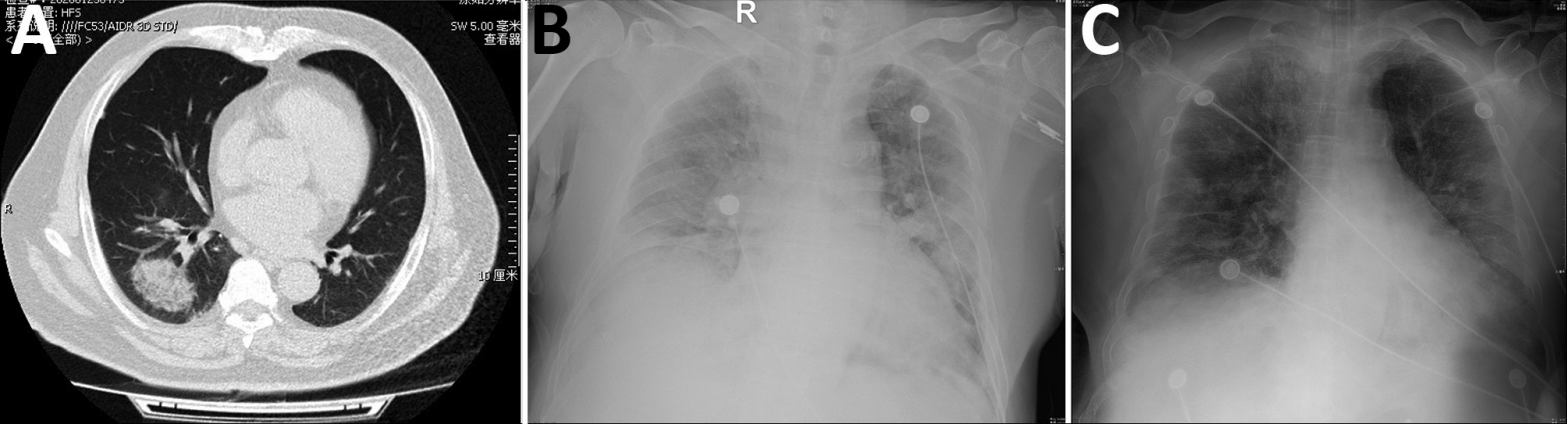
Radiographs of patient co-infected with severe acute respiratory syndrome coronavirus 2 and influenza A virus, China, 2020. A) Chest computed tomography demonstrating a mass, ground-glass consolidation in the right inferior lobe. B) Chest radiograph showing bilateral diffuse exudative shadows, indicating acute respiratory distress syndrome. C) Chest radiograph showing improved lung fields after 4 days in the intensive care unit.

We obtained a nasopharyngeal swab specimen and conducted real-time reverse transcription-PCR (rRT-PCR) for SARS-CoV-2 by using reagents provided by Shanghai BioGerm Medical Technology Co., Ltd. (http://www.bio-germ.com), and Da An Gene Co., Ltd. (Sun Yat-Sen University, http://en.daangene.com), on a LightCycler 480 (Roche, https://lifescience.roche.com). However, both tests returned negative results 8 hours later. We obtained another nasopharyngeal swab specimen for detection of SARS-CoV-2 and for differentiation of influenza A and B and respiratory syncytial viruses by using Xpert Flu/RSV Xpress assay (Cepheid, https://www.cepheid.com). The sample was negative for SARS-CoV-2 but positive for influenza A. The patient was discharged with oral oseltamivir and instructed to stay home for isolation.

On January 30, the patient returned to the hospital reporting persistent fever and aggravated dyspnea. Routine blood tests showed a leukocyte count of 8.23 × 10^9^ cells/L and lymphocyte count of 0.77 × 10^9^ cells/L. A chest radiograph showed diffuse exudative shadows in bilateral lungs, indicating acute respiratory distress syndrome (Figure, panel B). Physical examination revealed respiratory rate of 30 breaths/min and oxygen saturation of 83% on ambient air. We administered oxygen and screened another nasopharyngeal swab specimen, which was negative for SARS-CoV-2. Considering his clinical features, we performed a fourth test for SARS-CoV-2 by using a sputum sample, which also was negative. The patient’s dyspnea and respiratory distress increased, and his oxygenation index was <200. We admitted the patient to the single negative-pressure ward of the medical intensive care unit for severe influenza A pneumonia and administered endotracheal intubation because of severe hypoxemia.

Four days later, the patient’s oxygenation and chest radiographs improved (Figure, panel C). We performed a bronchoscopy and obtained bronchoalveolar lavage fluid (BALF) for metagenomic next-generation sequencing (mNGS) to identify potential pathogens. On February 5, mNGS reported 3,460 sequences that showed 99.8% identity and covered 98.69% of the SARS-CoV-2 genome NC_045512.2|SARS-CoV-2|Wuhan-Hu-1 (GenBank accession no. NC_045512.2). We then performed rRT-PCR by using newly collected sputum and stored BALF, which also tested positive. Cycle threshold values were 34 for sputum and 30 for BALF. However, a fourth nasopharyngeal swab collected concurrently with the second sputum sample remained negative. The next day, the patient was transferred to a designated hospital for further critical care.

This case highlights 2 challenges in the diagnosis of COVID-19. First, the sensitivity of tests to detect SARS-CoV-2 from upper respiratory specimens might be insufficient. Repeated rRT-PCR testing of nasopharyngeal swabs was negative for SARS-CoV-2 before the patient was admitted to the intensive care unit. To date, diagnosis of COVID-19 is made mainly on the basis of nucleic acid detection from nasopharyngeal swabs. For suspected cases, 2 negative findings from nasopharyngeal swabs performed ≥ 24 hours apart would exclude a COVID-19 diagnosis (*4*). In this case, without the clinicians’ persistence because of the patient’s travel history, a COVID-19 diagnosis might never have been established. SARS-CoV-2 finally was identified by using mNGS and rRT-PCR of a BALF sample. Therefore, suitable sputum or BALF specimens are necessary to maximize detection in cases of high clinical suspicion; mNGS also might be a helpful tool for identifying SARS-CoV-2 (*1*,*5*). 

Second, differentiating other causes of respiratory illness from COVID-19 is difficult, especially during influenza season, because common clinical manifestations of COVID-19, including fever, cough, and dyspnea, mimic those of influenza (*6*–*8*). In patients with COVID-19, blood tests typically show leucopenia and lymphopenia and most chest computed tomography scans show ground-glass opacity and consolidation with bilateral lung involvement (*7*–*9*). Unfortunately, influenza A and other respiratory viruses share these characteristics (*10*). Co-detection of SARS-CoV-2 and influenza A virus in this case demonstrates that additional challenges to detection remain, especially when patients test negative for SARS-CoV-2 but positive for another virus.

In summary, our case suggests that COVID-19 might be underdiagnosed because of false-negative tests for upper respiratory specimens or co-infection with other respiratory viruses. Broader viral testing might be needed when an apparent etiology is identified, particularly if it would affect clinical management decisions.
